# Evaluating Post-Treatment Effects on Electrospun Nanofiber as a Support for Polyamide Thin-Film Formation

**DOI:** 10.3390/polym16050713

**Published:** 2024-03-05

**Authors:** Anniza Cornelia Augusty, Ratthapol Rangkupan, Chalida Klaysom

**Affiliations:** 1 Center of Excellence in Particle and Material Processing Technology, Department of Chemical Engineering, Chulalongkorn University, Bangkok 10330, Thailand; annizagst@gmail.com; 2 Metallurgy and Materials Science Research Institute, Chulalongkorn University, Bangkok 10330, Thailand; ratthapol.r@chula.ac.th

**Keywords:** electrospinning, thin-film composite membrane, desalination, heat-pressed, hydrolysis

## Abstract

Poly(acrylonitrile-co-methyl acrylate) (PAN-co-MA) electrospun nanofiber (ENF) was used as the support for the formation of polyamide (PA) thin films. The ENF support layer was post-treated with heat-pressed treatment followed by NaOH hydrolysis to modify its support characteristics. The influence of heat-pressed conditions and NaOH hydrolysis on the support morphology and porosity, thin-film formation, surface chemistry, and membrane performances were investigated. This study revealed that applying heat-pressing followed by hydrolysis significantly enhances the physicochemical properties of the support material and aids in forming a uniform polyamide (PA) thin selective layer. Heat-pressing effectively densifies the support surface and reduces pore size, which is crucial for the even formation of the PA-selective layer. Additionally, the hydrolysis of the support increases its hydrophilicity and decreases pore size, leading to higher sodium chloride (NaCl) rejection rates and improved water permeance. When compared with membranes that underwent only heat-pressing, those treated with both heat-pressing and hydrolysis exhibited superior separation performance, with NaCl rejection rates rising from 83% to 98% while maintaining water permeance. Moreover, water permeance was further increased by 29% through n-hexane-rinsing post-interfacial polymerization. Thus, this simple yet effective combination of heat-pressing and hydrolysis presents a promising approach for developing high-performance thin-film nanocomposite (TFNC) membranes.

## 1. Introduction

Thin-film composite (TFC) membranes are commonly created using interfacial polymerization (IP) on a support layer as a prevalent method for producing effective membranes, particularly for water purification and desalination applications. TFC membranes typically consist of the following three major layers: the uppermost polyamide (PA) thin selective layer, the middle porous support layer, and the bottom non-woven fabric. The separation performance of the TFC membrane is largely dependent on the properties of the thin selective film, leading many research studies to focus on investigating the parameters influencing film formation, aiming to achieve and control the desired characteristics of the thin film [1,2,3,4,5]. Indeed, the formation of the PA-selective layer is significantly influenced by the properties of the support layer, such as pore size and hydrophilicity [6,7]. Conventionally, the support layer is produced using the phase-inversion method, but this technique is limited in terms of the support pore size range, often resulting in lower porosities and higher hydraulic resistances [8]. To address these limitations in phase inversion support, researchers have developed various modifications, including the addition of hydrophilic additives, such as polyvinyl pyrrolidone (PVP) [9,10], polyethylene glycol (PEG-400) [11] and titanium dioxide nanoparticles (TiO_2_) [12,13], as well as plasma treatment [14], to enhance the hydrophilicity and porosity of the hydrophobic support layer. Other studies applied 1D (carbon nanotubes/CNTs) and 2D (graphene oxide/GO) fillers [15,16] to provide additional water channel transport. Furthermore, the most recent strategy has been reported by incorporating the interlayer using the polyethyleneimine (PEI) interlayer [17] and 2D layered double-hydroxide (LDH) nanosheets [18] over the hydrophobic support layer to enhance membrane hydrophilicity and adhesion between the thin selective film and support, thus resulting in enhanced membrane selectivity.

An alternative to phase-inversion-produced support is the use of electrospun nanofibers (ENFs), which offer an interconnected pore structure, high porosity (>80%), large surface area, and ease of surface modification without the need for additional pore-forming agents [10,19]. However, the large and broadly distributed pore sizes of ENFs can lead to defects in the TFC membrane, including poor adhesion strength between the ENF support and the thin selective layer, potentially causing failure under high-pressure conditions.

Various research efforts have been undertaken to enhance membrane performances, such as modifying the top PA film using different monomers and fabricating the transition layer using chitosan [20], cellulose nanofiber [21], and polyethyleneimine (PEI) [22] onto the ENF support. Nonetheless, these strategies are complex and necessitate additional material processing, making them inefficient. Moreover, the additional transition layers can potentially decrease water permeability due to increased mass transfer resistance in the TFC membrane. There is a scarcity of studies focusing on support modification and its impact on PA film formation and overall membrane performance.

In this study, the PAN-co-MA copolymer was utilized because of its low cost, hydrophilic nature, good thermal stability, and great nanofiber spinnability [23,24,25]. While the PAN-co-MA copolymer has been extensively utilized in creating electrospun nanofibers for wastewater filtration applications [26], its application as a support layer for TFC membranes in desalination remains unexplored. A combination of heat-pressed treatment followed by alkali treatment was applied to ENF support before depositing the PA thin film. The heat-pressed treatment could enhance the mechanical integrity between the ENF support and the polyester non-woven back support, thus increasing the membrane’s strength [6,27]. The fiber diameter and support pore size can be tuned accordingly. The sodium hydroxide (NaOH) solution was utilized to alter the physicochemical and wetting properties of the nanofiber support through hydrolysis treatment [3]. This facile and efficient method is expected to produce thin-film nanofiber composite (TFNC) membranes with good water permeability and high salt rejection due to strong covalent and ionic bond interactions between the ENF support and PA film [28,29]. The effect of heat-pressed temperature and alkali hydrolysis temperature on the properties of the ENF support and TFC membrane was investigated. The performance of the obtained TFC membrane was examined and also compared to other studies.

## 2. Materials and Methods

### 2.1. Materials and Chemicals

Poly(acrylonitrile-co-methyl acrylate) containing copolymer acrylonitrile with 8.5% methyl acrylate (PAN-co-MA, Mw 150,000 g/mol, commercial grade) was provided by Haihang Industry (Hainan, China). N, N-dimethylformamide (DMF, 99.9%, reagent grade) was obtained from Carlo Erba (Cornaredo, Italy). Sodium dodecyl sulfate (SDS, ≥99%), triethylamine (TEA, ≥99.5%), and 1,3-phenylenediamine (MPD, 99%) were purchased from Sigma Aldrich (St. Louis, MO, USA). 1,3,5-benzenetricarbonyl chloride (TMC, 98%) was obtained from Acros Organics (NV, Geel, Belgium). N-hexane (analytical grade) was purchased from Anapure (Bangkok, Thailand). Sodium chloride (NaCl, 99%) was obtained from Ajax Finechem (Taren Point, NSW, Australia). Sodium hydroxide pellets (NaOH, 99%, Grade AR) were purchased from QReC (Auckland, New Zealand). Novatexx 2470 (polypropylene/polyester (PP/PE) non-woven backing support) was supplied by Freudenberg (Weinheim, Germany).

### 2.2. Fabrication of Electrospun Nanofiber Support

#### 2.2.1. Electrospinning and Heat Treatment

The electrospinning solution with a concentration of 8 wt.% was prepared by dissolving PAN-co-MA powder in DMF for 24 h at room temperature (25–28 °C) under continuous stirring to obtain a homogeneous solution. The polymer solution was injected into a syringe attached to a spinneret made of a 24G dull metallic needle. The solution was electrospun onto a PP/PE non-woven backing support, which was attached to the drum collector with a diameter of 7.5 cm and width of 35 cm. The PP/PE non-woven backing support was pre-wetted by dropping 5 mL of DMF onto the entire surface of the backing support before electrospinning to provide the adequate physical attachment of nanofiber webs to the basal region of the backing support. Electrospinning was carried out at an applied voltage of 23 kV, a flow rate of 1.50 mL/h with a 6 h duration, and a distance between the needle and the collector of 20 cm. After electrospinning, the obtained ENFs were air-dried at room temperature overnight to eliminate any residual solvent. The ENFs were sandwiched between two Teflon papers and heat-pressed at 120, 140, and 160 °C for 3 min (hereinafter labeled as hp-ENF 120, hp-ENF 140, and hp-ENF 160) using a commercial, compact fabric screen-printing machine (width 30 cm), equipped with an adjustable pressure knob, digital temperature, and time controls. It is worth mentioning that the pressure pressing on the ENF support was a controlled constant and equal to the weight of the top lid (10.36 kg).

#### 2.2.2. Hydrolysis Treatment

The hot-pressed nanofiber was immersed in a sodium hydroxide (NaOH) solution (2 M) at designated hydrolysis temperatures (30 °C and 50 °C) for 2 h. Then, the hydrolyzed nanofiber was rinsed with deionized (DI) water until the pH value of the residue reached neutral (pH~7). Afterward, the hydrolyzed nanofiber was dried in an oven at 60 °C for 10 min.

### 2.3. Fabrication of Thin Film Composite Membranes

**Figure 1:**
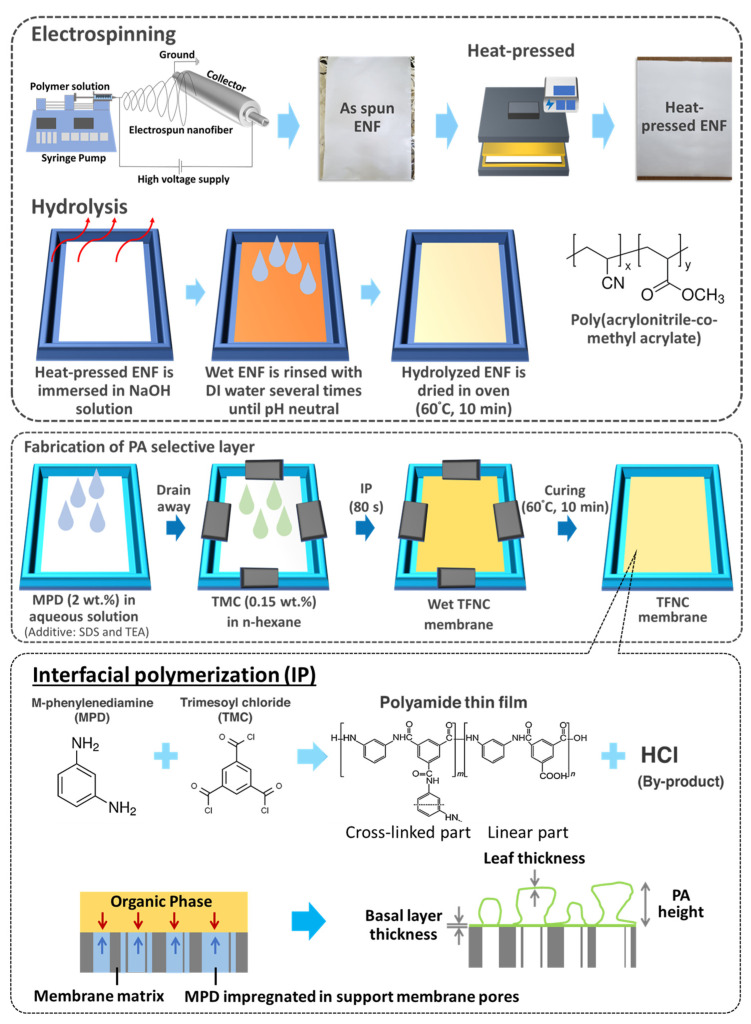
Schematic diagram of the fabrication of the electrospun nanofiber (ENF) support and thin-film nanofiber composite (TFNC) membranes via interfacial polymerization.

The polyamide (PA)-selective layer was fabricated on the ENF support layer via interfacial polymerization (IP) between the MPD and TMC monomers. The aqueous MPD solution was prepared by adding 2 wt.% of MPD flakes, 2 wt.% of TEA, 0.1 wt.% of SDS into 95.90 wt.% of deionized (DI) water under a stirring condition at room temperature. The TMC organic solution was prepared by dissolving 0.15 wt.% of TMC into n-hexane. At room temperature, the amine aqueous solution was introduced to the electrospun nanofiber for 30 min. The excess solution was removed after draining the amine aqueous solution by gently wiping the surface of the ENF support with tissue paper, and it was left to dry partially under a fume hood for 80 s at room temperature. The amine-saturated ENF support was mounted on the gasket frame and held with clamps on each edge to deter the solution leakage during the reaction process. A PA thin film was deposited on the ENF support layer after the TMC solution was poured over the reaction surface area to conduct the IP reaction for 80 s. The TMC solution was drained out, and the PA TFNC membrane was subsequently and thermally cured in an oven at 60 °C for 10 min. The prepared PA TFNC membrane was immersed in DI water until further use. For the PA TFNC membrane with an n-hexane rinse, 30 mL of n-hexane was poured onto the ENF support, and the TFNC membrane was rinsed for 5 s after the IP reaction. The rest of the protocols were followed, as previously mentioned. The schematic diagram of the fabrication of TFNC membranes is depicted in Figure 1. The PA TFNC membrane prepared on untreated ENF is labeled as pristine TFNC, and TFC membrane prepared on heat-pressed nanofibers (hp-ENF) is labeled as hp-TFNC, and the membrane with heat-pressed and hydrolyzed nanofibers (hp-ENF x) is referred to as TFNC y, where x and y refer to the heat-pressing temperature and hydrolysis temperature, respectively.

### 2.4. Material Characterization

#### 2.4.1. Scanning Electron Microscopy (SEM)

The surface morphology of the ENF support and PA TFNC membrane was observed using scanning electron microscopy (SEM, Hitachi S-3400N, Tokyo, Japan) with a secondary electron detector. Observations were conducted at an accelerating voltage of 15 kV, achieving a resolution of up to 100 nm. Image-J software (Java 8, National Institutes of Health, Bethesda, MD, USA) was used for analyzing the averaged fiber size and size distribution. The cross-section morphology for the PA TFNC membrane was also observed.

#### 2.4.2. Pore Size Measurement

The pore diameter and pore size distribution of the ENF support were characterized by a capillary flow porometer (Porolux 1000 series, Aptco Technologies, Nazareth, Belgium). The bubble point pressure method was used to determine the pore size.

#### 2.4.3. Fourier-Transform Infrared Spectroscopy (FTIR)

The chemical composition of the ENFs and TFNC membranes was analyzed by attenuated total reflectance-Fourier transform infrared spectroscopy (ATR-FTIR, Thermo Scientific, Nicolet iS50 spectrometer, Waltham, MA, USA) at a scanning range of 400–4000 cm^−1^ (Diamond crystal, resolution 4 cm^−1^).

#### 2.4.4. Contact Angle

The membrane surface hydrophilicity was evaluated at room temperature by a contact angle goniometer (Dataphysics, OCA-25, Filderstadt, Germany) with a sessile drop method using 5 µL of DI water.

#### 2.4.5. X-ray Photoelectron Spectroscopy (XPS)

The elemental compositions and their chemical states on the ENF support and TFNC membrane were analyzed using XPS (Kratos Axis Ultra DLD, Manchester, UK) with Al-K alpha as an X-ray source (10 mA, 15 kV). The O/N ratio of the PA thin film can be measured from atomic compositions obtained by XPS.

#### 2.4.6. Confocal Laser Scanning Microscopy (CLSM)

The top surface of TFNC membranes was examined using CLSM (Olympus LEXT OLS5000, Tokyo, Japan) to visualize 2D and 3D morphologies with a scanned area of 644 µm × 648 µm. The lateral scanning detection was performed by locating the area with an edge boundary between each region of nanofiber support and PA thin film with optical images captured at 20× and 50× magnification. The image profile was generated by the Olympus OLS5100 data acquisition application.

#### 2.4.7. Mechanical Strength Testing

The mechanical properties of TFNC membranes were measured with a universal testing machine (ZwickRoell ProLine, Ulm, Germany) with a sample size of 60 × 10 mm, a load cell of 500 N, and an elongation rate of 1.0 mm/s. The specimens were tested according to the ASTM D1708 standard [30].

### 2.5. Membrane Performance Evaluation

Water permeance and NaCl rejection were evaluated by a crossflow filtration membrane module (Sterlitech CFO16D-CF, Auburn, WA, USA) at 5 bars with a membrane effective area of 14.44 cm^2^. Sodium chloride (NaCl, 2000 ppm) was fed to the system at 0.8 L min^−1^. The salt concentration in the feed and permeate side was measured by a conductivity meter (Metler Toledo, FiveEasyTM FE30, Columbus, OH, USA). The water flux and water permeance (or pressure-normalized flux) were estimated by Equations (1) and (2), respectively [31].

(1) $$J_{W}=\frac{V}{A_{M}\;∆t}$$

(2) $$A=\frac{J_{W}}{(∆P-∆\pi )}$$

where J_W_ is the water flux (Lm^−2^h^−1^ or LMH), V is the volume of permeated water (L), A_M_ is the effective area of the membrane (m^2^), Δt is the permeation time (h), A is the pressure-normalized flux or water permeance (Lm^−2^ h^−1^bar^−1^ or LMH bar^−1^), ΔP is the transmembrane pressure (bar), and Δπ is the feed osmotic pressure difference (bar).

The salt rejection (R, %) was calculated by Equation (3).

(3) $$R=\left(1\;-\frac{C_{P}}{C_{F}}\right)\times \;100$$

where C_F_ is the salt concentration of feed (ppm), and C_P_ is the salt concentration on the permeated side (ppm). 

### 2.6. Membrane Stability Testing

Using the same setup and procedure described in Section 2.5, the performance of the developed membranes over a longer operating duration was examined to evaluate the stability of the TFNC membrane over 24 h.

## 3. Results and Discussion

### 3.1. The Effect of Heat Treatment on the Support Properties

**Figure 2:**
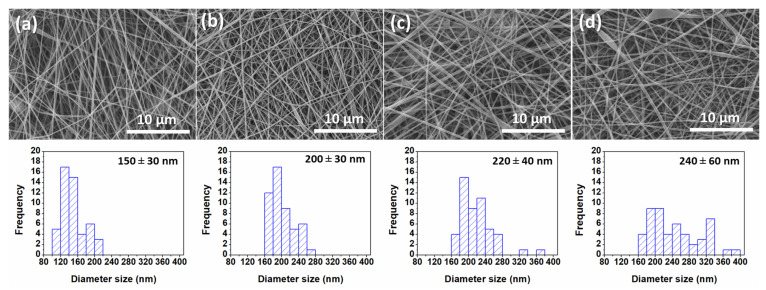
SEM images and fiber size distribution of (**a**) As-spun ENF, (**b**) hp-ENF 120, (**c**) hp-ENF 140, and (**d**) hp-ENF 160.

The SEM surface morphology of the ENFs before and after heat-pressing is shown in Figure 2. From the SEM images, it was observed that the average fiber diameter increased from 150 nm to around 220 nm after the heat treatment. The ENF support appeared to be more compact and denser after heat-pressing, creating a stable and strong inter-fiber connection and preventing the fibers from slipping under high-pressure operation [6]. Moreover, it was also observed that the nanofiber could attach well to the backing materials, making them easier to handle compared to the untreated ones. The higher the temperature, the flatter the fibers [32]. The opening between the fibers was, thus, reduced due to the heat-induced contraction of the nanofibers [33]. Consequently, the mean pore size of the ENF support was significantly decreased with heat press temperatures, contributing to an increase in the pressure required for determining the mean pore size (Table 1). It was speculated that the reduction in pore size could be attributed to both nanofiber flattening and partial melting occurring in some areas of the PP/PE non-woven backing support (See Figure S1). Similar observations were also reported by Wu et al. and Yao et al., where smaller pore sizes were obtained after applying the heat-pressed treatment on electrospun PVDF nanofibers [32,34].

**Figure 3:**
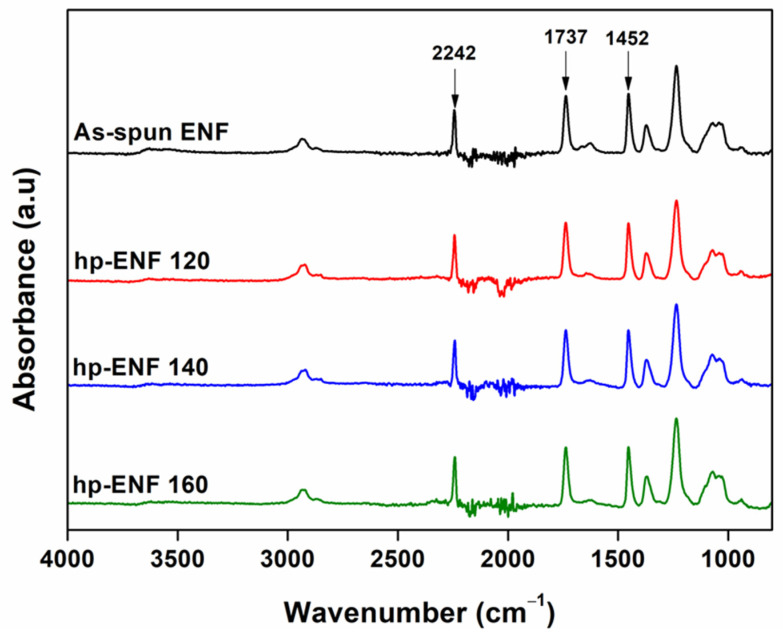
FTIR spectra of ENF support for different heat-pressed temperatures.

To check the change in the chemical structure of ENF after the heat treatment, the ATR FTIR analysis was carried out, as depicted in Figure 3. The typical peaks of PAN-co-MA nanofibers without any sign of chemical structure change after thermal treatment were observed in all samples. The stretching vibration of C≡N (nitrile group) and bending of -CH_2_ (methylene group) was detected at 2242 cm^−1^ and 1452 cm^−1^, respectively [35], whereas the peak at 1737 cm^−1^ was determined by the carbonyl bonds of the methyl acrylate co-monomer [36,37]. This indicated that the heat-pressed treatment in the experimental range of 120–160 °C on the ENF support only allowed physical alteration, but no chemical decomposition was involved.

### 3.2. Effect of Heat Treatment on Thin-Film Formation and Membrane Performance

**Figure 4:**
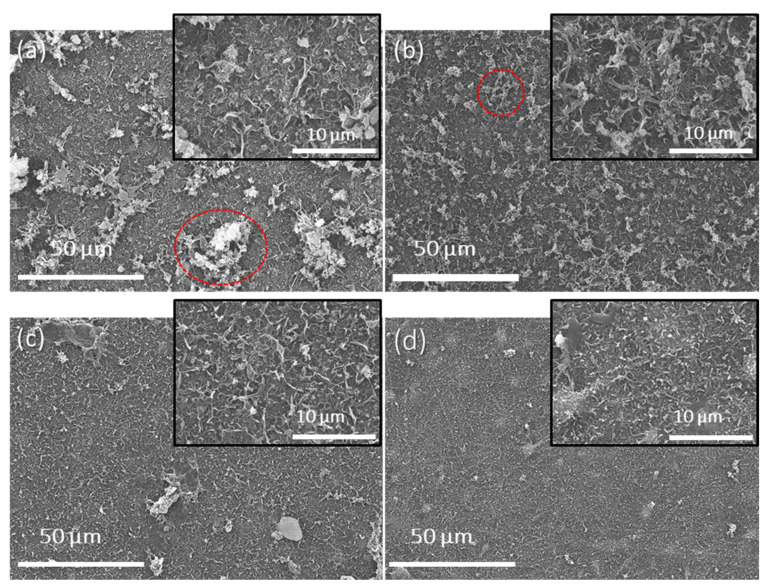
SEM images of (**a**) pristine TFNC, (**b**) hp-TFNC 120, (**c**) hp-TFNC 140, and (**d**) hp-TFNC 160 at 1000× magnification (insert pictures: 5000× magnification).

The TFNC membranes were successfully fabricated by interfacial polymerization, forming a PA thin selective layer over the prepared ENF support. The SEM images in Figure 4 illustrate the top surface of these membranes.

A characteristic ridge-and-valley structure is evident across all samples of the ENF support. The pristine thin-film nanocomposite (TFNC) membranes exhibited a rougher surface with prominent clusters of polyamide (PA) lumps, as indicated by the red circle in Figure 4a. In comparison, the TFNC membranes fabricated with a heat-pressed electrospun nanofiber (ENF) support presented a significantly smoother surface. When ENF supports with larger pore sizes were used (for instance, as-spun ENF with a pore size of 1.26 µm), MPD rapidly diffused toward the TMC organic phase. This process is primarily governed by Marangoni convection, which leads to a larger contact area for the reaction zone and, consequently, rapid film growth [1,9]. As a result, the PA films formed under these conditions are characterized by larger globular structures and a rougher texture. Conversely, when the support has smaller pores, the movement of MPD toward TMC is hindered, resulting in the formation of a smoother surface with smaller PA film structures. This explanation is consistent with Li et al. [2], who used hydrophilic supports made from cellulose acetate propionate, fabricated via phase inversion, for interfacial polymerization to create PA TFC membranes. Similarly, Han et al. [28] documented the uniform formation of a PA-selective layer on a hydrophilic PAN nanofiber support with pore sizes (0.53–0.94 µm) that are comparable to those in our study. Despite the relatively limited research into the effects of the hydrophilic ENF support pore size on PA film formation, it is notable that the ENF supports produced in the current study, with much larger pore sizes than those made by conventional phase inversion supports (ranging from 10 to 150 nm) [2,9,38], can still facilitate the development of a uniform, defect-free PA-selective film.

**Figure 5:**
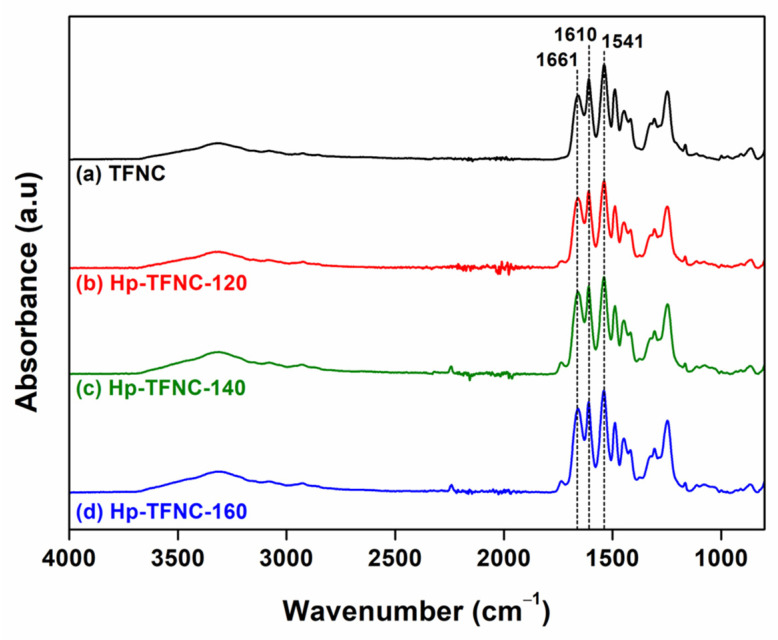
FTIR spectra of hp-TFNC membranes.

To verify the formation of PA layers on an ENF support, ATR-FTIR was used to examine the chemical bonding, as shown in Figure 5.

All TFNC membranes exhibited an amide characteristic peak, and there were three main peaks at 1661 cm^−1^ (C=O stretching, amide I), 1610 cm^−1^ (C=O from hydrogen bond, amide I), and 1541 cm^−1^ (bending of N-H and stretching vibration of -CN, amide II) [3]. These peaks represented the successful formation of cross-linked and an aromatic dense PA-selective layer on the ENF support. It is worth mentioning that the low-intensity absorption bands at 2242 cm^−1^ and 1737 cm^−1^, which are the characteristics of the underlying ENF support, become more pronounced in the samples subjected to heat treatment. Notably, after heat pressing, the PA film on the support becomes smoother with fewer clusters of PA lumps, as evidenced by the SEM images (Figure 4). This transformation likely leads to the thinner layer of the PA film. As a result, the FTIR beam is able to penetrate deeper into the ENF support layer, making the detection of these specific absorption bands more pronounced, especially in the heat-treated supports. This enhanced detectability may also be ascribed to the densification of the ENF support post-heat-pressing, which amplifies the likelihood of these bands being detected.

**Figure 6:**
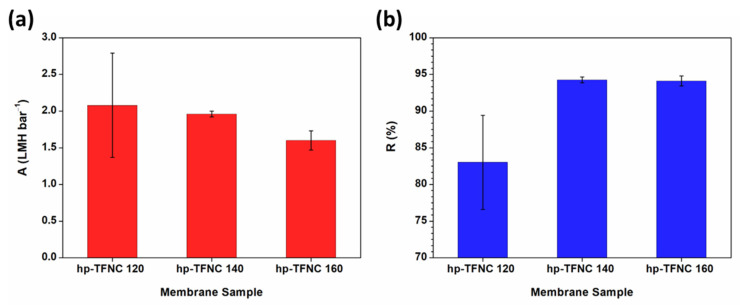
(**a**) Pressure-normalized flux (A) and (**b**) NaCl rejection (R) of TFNC membranes prepared from ENF supports with different heat-pressed temperatures.

The performance of the TFNC membrane prepared from ENF supports heat-pressed at different temperatures was determined. The prepared membranes demonstrated an improved NaCl rejection as the temperature of heat pressing increased, although there was a corresponding decrease in permeance, as illustrated in Figure 6. This outcome presents a trade-off between enhanced salt rejection and a reduced pressure-normalized flux. A possible explanation for the observed decrease in pressure-normalized flux could be justified by the reduction in pore size of the ENF support, which leads to increased mass transfer resistance, as suggested by previous reports [32,39]. Kaur et al. explained an identical trend in which the lower water flux of the TFC membrane was associated with smaller pore sizes of nanofiber support [6]. Additionally, the partial melting of non-woven backing materials at elevated temperatures (Figure S1) could contribute to diminished water permeance. This is supported by the significant drop in water permeance of the backing support after being heat-pressed at 140 °C, as detailed in Table S1. Increasing the heat-pressed temperature, on the other hand, improves the TFNC membrane’s selectivity over monovalent salt [40], with improvements of up to 94%. This indicates the more effective formation of a dense polyamide (PA)-selective film. It should be noted that the TFNC membrane prepared on an untreated ENF support performed poorly in separation and exhibited no selective characteristics. To avoid the additional mass transfer resistance caused by partial melting, the ENF treated at 120 °C was chosen for further hydrolysis modification.

### 3.3. The Effect of Combined Heat Treatment and Hydrolysis on Support Properties

**Figure 7:**
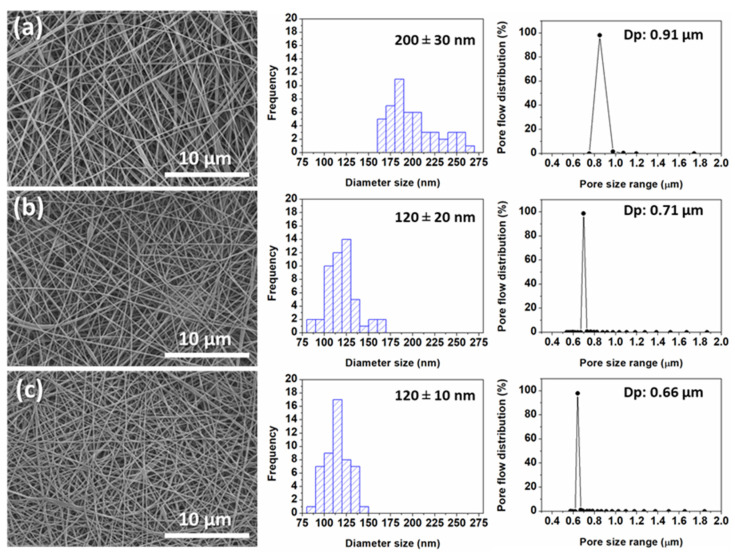
SEM images, fiber size distribution and pore size of hp-ENF 120 (**a**) before hydrolysis and after hydrolysis with 2 M of NaOH for 2 h: (**b**) 30 °C and (**c**) 50 °C.

The hp-ENF 120 support layer was treated with 2 M of NaOH solution at 30 and 50 °C for 2 h. From Figure 7, after hydrolysis, it can be seen that there was a marked reduction in the average fiber diameter, which shrank from approximately 200 nm to around 120 nm. 

A similar observation was reported by Han et al. [28]. A previous study by Ilyas et al. reported similar findings and suggested that the reduction in fiber size could be attributed to the removal of amorphous regions within the nanofibers during NaOH hydrolysis [41]. However, in our study, the weight loss observed after hydrolysis was negligible (<0.1%) (see Table S2). Therefore, it is possible that the decrease in fiber size was due to the relaxation of fibers that had previously been flattened. Additionally, the supports became denser and more compact due to fabric shrinkage, leading to an increase in fiber density [42,43]. Consequently, the pore size of the electrospun nanofiber (ENF) support experienced a reduction of about 20–28% following hydrolysis, as detailed in Table 2. This might also result in the improved entanglement degree of the modified ENF after hydrolysis [44,45]. Electrospun nanofiber film shrinkage during drying typically occurs when the solvent in the nanofiber film evaporates, causing the film to contract and shrink. The degree of shrinkage of the film was also reported to be influenced by many factors, including the wettability of the film.

**Figure 8:**
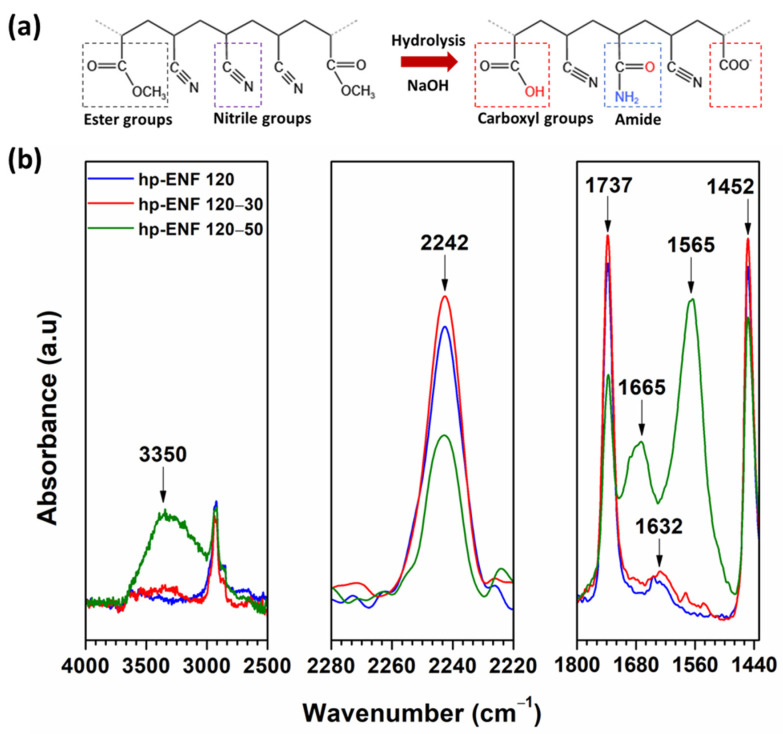
(**a**) Possible reaction mechanism of PAN-co-MA hydrolysis and (**b**) FTIR spectra of hp-ENF 120 before and after hydrolysis at 30 °C (hp-ENF 120-30) and 50 °C (hp-ENF 120-50).

In the hydrolysis process of PAN-co-MA nanofibers, the nitrile groups (-CN) were initially targeted by NaOH molecules. This resulted in the following two-stage transformation: first into amide (-CONH_2_) and then into carboxylic acid (-COOH) [46]. This hydrolysis process of the hp-ENF and NaOH is evident in the FTIR spectra (Figure 8). The nitrile group’s peak at 2242 cm^−1^ and the ester bond’s peak at 1737 cm^−1^ decreased in intensity, while the intensities of the amide (at 1665 cm^−1^ and 1632 cm^−1^) and carboxylic acid (at 1565 cm^−1^) peaks increased following hydrolysis. Furthermore, a new, significant peak appeared at 3350 cm^−1^, which corresponded to the -OH bond in carboxylic acid [47]. Additionally, increasing the temperature during the hydrolysis reaction can speed up the creation of -CONH_2_ and -COOH groups as a result of more extensive hydrolysis [48]. Specifically, at a temperature of 50 °C, the reaction became faster, leading to the formation of additional hydrophilic functional groups. This is evidenced by the appearance of new chemical bonding peaks. The proportions of -CONH_2_ and -COOH relative to the -CN groups have been precisely calculated and are detailed in Table 2. Notably, the ratios of -CONH_2_/-CN and -COOH/-CN in the sample hydrolyzed at 50 °C are greater than those in samples treated at 30 °C or in non-hydrolyzed ENF. This indicates the more significant degree of hydrolysis, signifying enhanced hydrophilic and wetting properties of the hp-ENF support, which is a fact further supported by the results of the contact angle test (Figure S2). After hydrolysis, the water contact angle of the support reduced from 25° to 0°.

**Figure 9:**
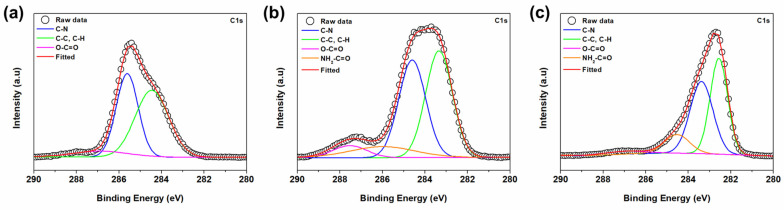
High-resolution C1s XPS spectra of (**a**) hp-ENF 120, (**b**) hp-ENF 120-30, and (**c**) hp-ENF 120-50 support layer.

X-ray Photoelectron Spectroscopy (XPS) was employed to analyze the chemical structural changes in the PAN-co-MA nanofiber support following hydrolysis. Figure 9 illustrates the high-resolution C1s deconvolution of the PAN-co-MA nanofiber both pre- and post-hydrolysis. Initially, the C1s peak for the non-hydrolyzed nanofiber displayed three distinct peaks at 284.4, 285.6, and 286.9 eV, corresponding to the -C-C and -C-H, -CN, and -CO groups, respectively (as shown in Figure 9a) [49]. Post-hydrolysis, new peaks were observed at 286 and 287.6 eV, indicative of the formation of amide (-CONH_2_) and carboxyl (-COOH) groups (refer to Figure 9b) [50]. Upon increasing the hydrolysis reaction temperature, a shift in the entire spectrum to lower binding energies was noted, coupled with a reduction in the intensity of the deconvoluted -CN peak. Conversely, the intensities of the deconvoluted amide and carboxyl group peaks increased (see Figure 9c). This XPS analysis, corroborated by previous Fourier-transform infrared spectroscopy (FTIR) findings, confirms the partial conversion of -CN groups into -CONH_2_ and -COOH groups. Moreover, elevating the reaction temperature further enhances the formation of amide and carboxyl groups.

### 3.4. The Effect of Combined Heat-Pressing and Hydrolysis on Thin-Film Formation and Membrane Performance

**Figure 10:**
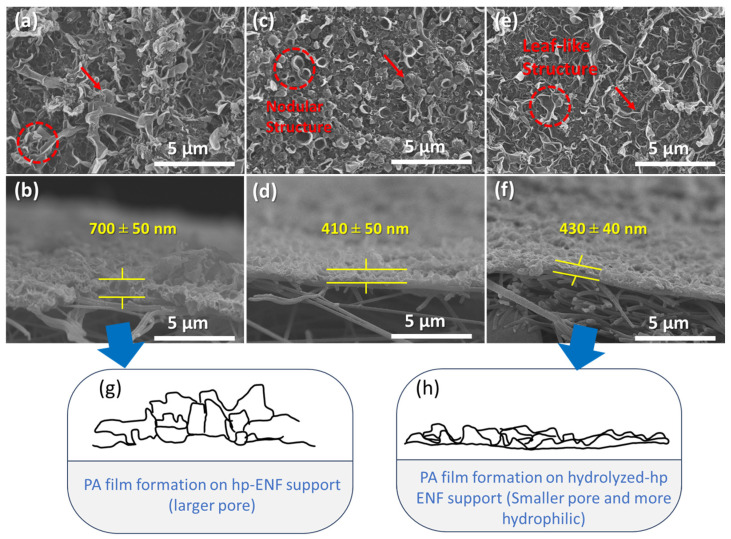
SEM images of the top surface and cross-section TFNC membranes prepared on (**a**,**b**) non-hydrolyzed ENF and hydrolyzed hp-ENF 120 at (**c**,**d**) 30 °C and (**e**,**f**) 50 °C with 2 M of NaOH solution for 2 h. (**g**,**h**) Represent the sketch of the PA film deposited on the support with and without hydrolysis treatment, respectively.

The surface and cross-section morphology of TFNC membranes were characterized. As represented in Figure 10, the PA-selective films, applied to all ENF supports, exhibited a characteristic ridge-and-valley structure interspersed with leaf-like formations (see red arrows). However, significant variations were observed in the surface morphology of the TFNC membranes (see red circles). These differences were attributed to the variations in pore size and hydrophilicity of the ENF supports. Given that the polyamide thin selective layer was synthesized following the same procedures, significant variances could be explained by differences in the ENF support pore size and hydrophilicity.

Specifically, the PA film applied to a non-hydrolyzed ENF support (Figure 10a) demonstrated predominantly leaf-like structures with a loosely arranged multi-layered PA configuration. In contrast, the PA film on hydrolyzed ENF support at 30 °C (Figure 10c) showed a greater abundance of small nodules and dense, crumpled bumps. This difference was likely due to alkaline hydrolysis, which resulted in the filling of smaller support surface pores with an amine-aqueous solution. This process slowed the migration of MPD molecules, thereby limiting their convection towards the organic phase due to the restricted amount of MPD absorbed in the pores. As a result, the resultant PA thin film had a higher concentration of small nodules. Conversely, larger support pore sizes enhanced the swift migration of MPD towards the reaction interface through Marangoni convection. This unstable convection led to an expanded reaction contact area, facilitating rapid film growth characterized by an extensive ridge-and-valley structure [2,15,51].

Upon increasing the hydrolysis reaction temperature to 50 °C, there was a further reduction in the pore size of the support structure, as detailed in Table 2. A smoother and relatively thinner film (thinner than that of the non-hydrolyzed one) was observed; however, unexpectedly, a none-nodular structure was observed (Figure 10e). This observation contrasts with the trends reported in previous studies using phase-inversion support layers [4,9,52], where the opposite effect was noted. This might be attributed to the negatively charged functional groups (-COOH) in the ENF support that were significantly generated after hydrolysis at 50 °C. These negatively charged functional groups can alter the diffusion rate of MPD to the reaction zone and result in a different film structure. A similar phenomenon was noted by Liu et al., where polyethersulfone (PES) support, functionalized with carboxylate cellulose nanocrystals, was used in fabrication [53].

The PA film’s thickness could also be estimated from the cross-section morphological images shown in Figure 10. It is shown that the PA-selective layer seems to firmly attach to the underlying ENF support. According to the cross-section images, the thickness of the PA film decreased from approximately 700 nm (as seen in Figure 10b) to around 400 nm (Figure 10d,f) once the supports were hydrolyzed. This observation is in agreement with the findings reported by Park et al. and Ghosh et al. [3,54]. As hydrolysis is carried out, the ENF support attains a superhydrophilic state. This transformation, coupled with the smaller pore sizes, creates conditions that are conducive to forming the PA layer predominantly at the membrane surface [55,56]. The hydrophilic ENF support enables the slow MPD migration toward the reaction platform to react with the TMC solution, and with a small support pore size, the absorbed MPD remains trapped inside it [52]. Additionally, in the case of the non-hydrolyzed ENF support, the PA thin film exhibits voids that appear to merge with adjacent PA layers. During the interfacial polymerization reaction, the MPD solution (in large-pore support) potentially migrates rapidly near the water–hexane interface to react with the TMC monomer. This leads to the pushing away of the initially formed PA layer and the formation of a large film pocket [3,9]. The rapid IP reaction is an exothermic reaction, releasing heat and producing hydrochloric acid (HCl), which, in turn, allows nanobubbles to form [57]. These nanobubbles are inherently unstable and tend to burst, releasing from the formed PA film and resulting in a looser PA film characterized by a leaf-like structure [7]. Furthermore, the formation of a defective PA film is a notable issue, likely induced by the larger pore sizes of the non-hydrolyzed TFNC membranes. Such imperfections may account for the considerable variability in permeance and the diminished sodium chloride (NaCl) rejection observed in the next sections. This is attributed to the non-uniform distribution of the PA film across the ENF support. To further confirm the hypothesis and to understand the mechanism of thin-film formation, its detailed in situ characterization during the film formation is recommended.

**Figure 11:**
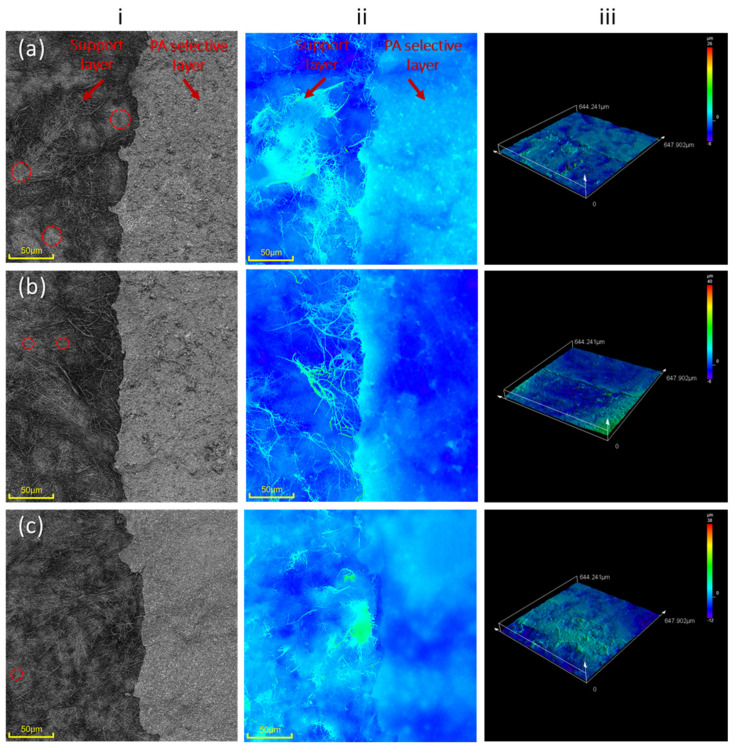
CLSM surface images of (**a**) hp-TFNC 120, (**b**) hp-TFNC 120-30, and (**c**) hp-TFNC 120-50 membrane with (**i**) 2D optical view and high intensity, and (**ii**,**iii**) 2D- and 3D-mapped topography of TFNC structure, respectively.

Despite this unexpected variation, the TFNC membrane from hydrolyzed support at 50 °C showed better adhesion of the PA thin film to the support compared to its non-hydrolyzed counterpart. This is evident in Figure S3, which shows the stronger attachment of the PA film on the hydrolyzed hp-ENF 120 support when tested with double-sided tape. Such robust adhesion between the PA layer and the ENF support contributed to the improved performance of the membrane. To further validate the adhesion between the PA layer and the support, confocal laser scanning microscopy (CLSM) was employed to capture the optical images of the membrane’s top surface across its depth. Figure 11 shows distinguishable patterns of the nanofiber support layer (left side) and PA-selective layer (right side) after being removed from the double-sided adhesive tape. Some imprints of PA-selective film (indicated by red circles) were still visible on the peeled TFNC surface. However, a portion of the thin selective layer covering the nanofiber was diminished, leaving the fiber structure on the half side of hp-TFNC 120-30 and hp-TFNC 120-50. This proves that the alkali hydrolysis treatment could improve the attachment of PA-selective film on ENF support. In addition, the 2D top-most surface of all TFNC membranes revealed a smoother PA-selective layer at a higher hydrolysis temperature (hp-TFNC 120-50), which correlated with the SEM result.

**Figure 12:**
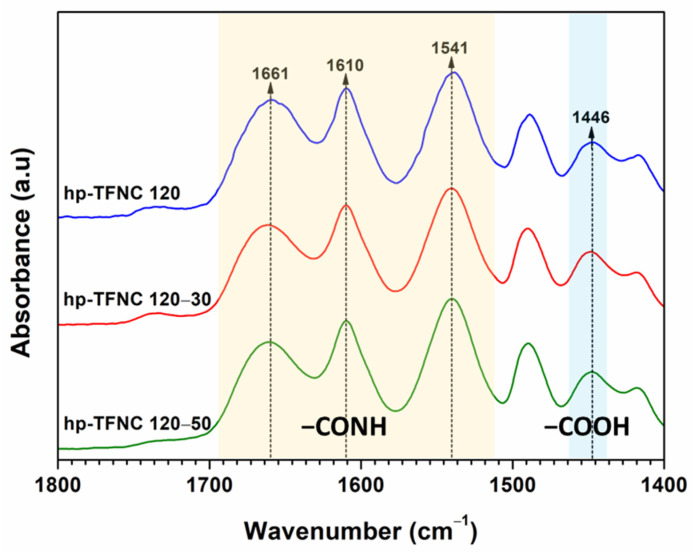
FTIR spectra of TFNC membranes prepared on hydrolyzed ENF at different temperatures.

The characteristic chemical structure of the PA film at 1661 cm^−1^, 1610 cm^−1^, and 1541 cm^−1^ was confirmed, as shown in Figure 12. Accordingly, the C-O stretching of the linear COOH groups reached 1446 cm^−1^ due to the hydrolysis of acyl chloride, contributing to the linear structure of the PA-selective film [58]. Besides the evidence of PA formation, the peak intensity ratios between the amide and carboxyl groups varied across all TFNC membranes. To specify the values, the ratio of -COOH (from linear PA)/-CONH (from cross-linked PA) could be determined by defining the area under the curve from FTIR spectra, and these are compared in Table 3.

**Figure 13:**
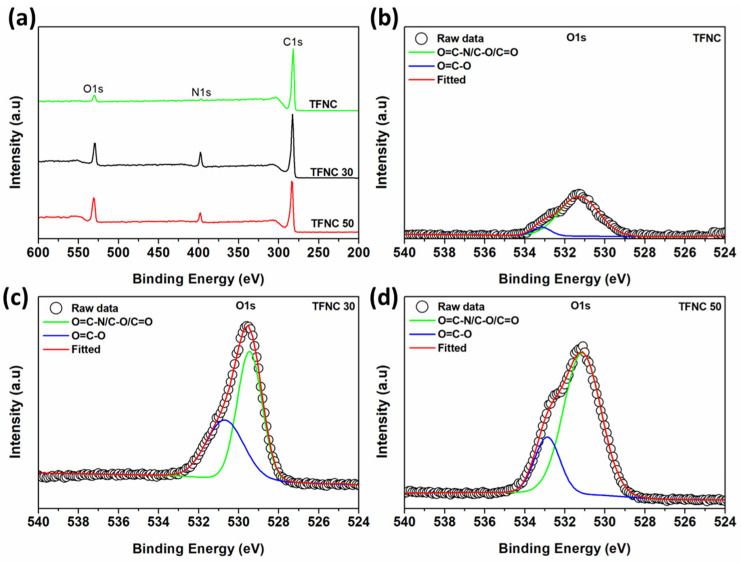
XPS spectra of TFNC membranes: (**a**) wide scan, O1s high resolution of (**b**) TFNC before hydrolysis, (**c**) TFNC 30 and (**d**) TFNC 50.

A higher -COOH/-CONH (I_1446_/I_1541_) ratio indicates that more acyl chloride was hydrolyzed with water molecules, forming a more linear form of the PA film. This can affect the separation performance of the membranes [59]. The results disclosed that the stronger the alkaline hydrolysis, the higher the ratio of -COOH/-CONH in the PA layer. The surface elemental composition of TFNC membranes was also determined by XPS, as illustrated in Figure 13. From the O1s spectra of the prepared TFNC membranes (Figure 13b–d), the two characteristic peaks were noticed at 531.2 and 532.9 eV, which belong to the -CONH and -COOH groups, respectively [60]. The O/N ratio estimated from XPS is typically used to determine the crosslinking degree of the PA film, with typical O/N values ranging from 1 to 2. The O/N ratio close to 1 indicates a highly cross-linked PA structure [61]. However, it was observed that the O/N ratio for some samples surpassed the upper limit of this range. It has been suggested that relying on the O/N ratio from XPS to estimate the crosslinking degree of PA might lead to inaccuracies. This is because the detected oxygen component could originate from sources other than the sample itself, and the O/N ratio is notably susceptible to the method of sample preparation [62,63].

It is important to recognize that the trends identified in the linear and cross-linked segment ratio of the polyamide (PA) film, as determined by FTIR and XPS, differ. The FTIR analysis suggests that the stronger hydrolysis of the support correlates with an increase in linear PA segments. On the other hand, the O/N ratio of non-hydrolyzed thin-film nanocomposite (TFNC) membranes, as indicated by XPS spectra, does not align with this trend and is, in fact, contradictory to the FTIR results. This discrepancy may stem from the different penetration depths of the two analytical techniques. The penetration depth of FTIR varies with the wavelength. Specifically, in the characteristic region for PA, between 1400 and 1700 cm^−1^, the FTIR beam can penetrate deeper than 1 µm. This depth exceeds the thickness of the PA film, allowing the beam to reach the underlying ENF support. In contrast, XPS has a significantly shallower penetration depth, limited to only 2–10 nm into the PA layer [64]. The presence of linear structures, which include carboxyl groups in the PA, is well recognized for imparting a more hydrophilic nature to the film, which is advantageous for water permeance.

**Figure 14:**
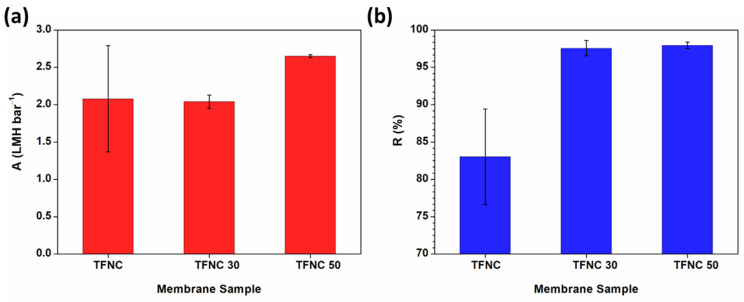
(**a**) Pressure-normalized flux (A) and (**b**) NaCl rejection (R) of TFNC membranes prepared on non-hydrolyzed ENF and hydrolyzed ENF at different hydrolysis temperatures.

The separation performance of the fabricated membranes was assessed at 5 bars using 2000 ppm of the NaCl solution, as depicted in Figure 14. Both permeance and the NaCl rejection of TFNC membranes deposited on hydrolyzed hp-ENF support were enhanced, implying that hydrolysis modification had a positive impact on the film formation. The improved water permeance of TFNC from the hydrolyzed support was attributed to the much thinner and more hydrophilic selective film. These results are also in good agreement with the high ratio of -COOH/-CONH from FTIR previously discussed. Additionally, the large variation in the separation performance of the TFNC membrane from non-hydrolyzed support could be due to the uneven film formation and less effective adhesion of the film to the support, potentially leading to defects.

The NaCl rejection of TFNC membranes was significantly enhanced by 15% after ENF support was treated by an alkaline, maintaining a value above 95%. The remarkable salt rejection can be attributed to the reduction in the support pore size following hydrolysis, resulting in a more uniform and compact thin film. Additionally, the improvement in performance might be due to enhanced interactions between the support and the PA film, characterized by strong ionic and covalent bonding, as well as increased hydrophilicity [29,65,66]. The underlying mechanism of this enhancement is further elaborated in the Supplementary Materials (Figure S4).

**Figure 15:**
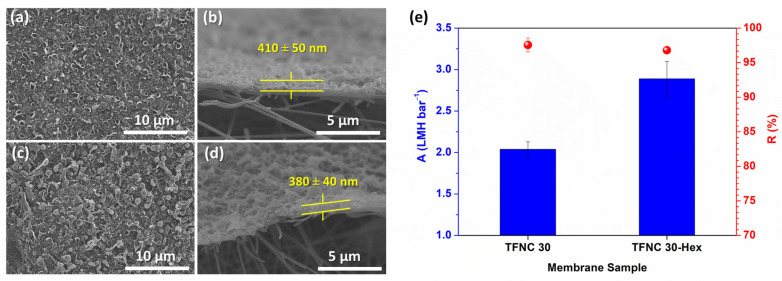
SEM images of top-surface and cross-section TFNC membranes prepared on hydrolyzed hp-ENF with 2 M of NaOH at 30 °C for 2 h (**a**,**b**) without n-hexane rinsing and (**c**,**d**) n-hexane rinsing after IP reaction. (**e**) Performance comparison.

To further enhance the pressure-normalized flux, an n-hexane rinse is applied after the IP reaction during the PA formation stage. It is revealed that the pressure-normalized flux was greatly enhanced while retaining high salt rejection at about >95% (Figure 15) compared to TFNC without an n-hexane rinse. The n-hexane rinse is beneficial to residual TMC monomers, creating a thinner PA-selective layer [67]. Based on XPS analysis (Figure S5), after the n-hexane rinse, the O/N ratio dropped while the amide/carboxyl ratio increased (Table S3). This led to an enhanced pressure-normalized flux without sacrificing the NaCl rejection (summary in Table 3). This remarkable result was consistent with the previously reported paper [68].

### 3.5. Performance Stability and Comparison

**Figure 16:**
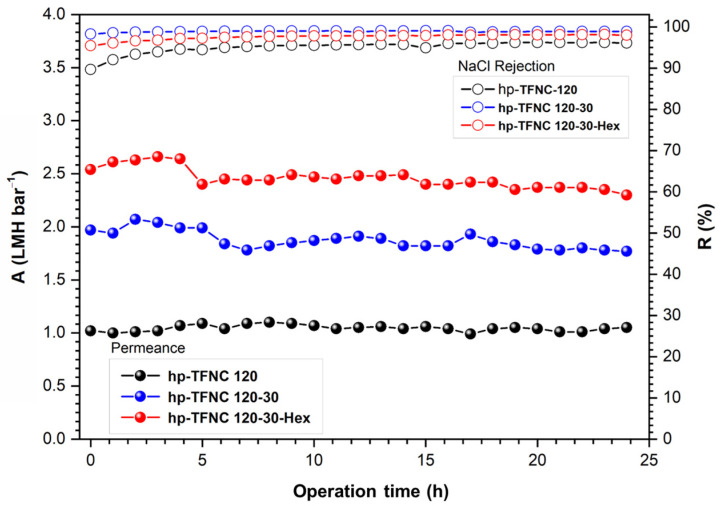
Stability performance of TFNC membranes before and after hydrolysis and after n-hexane rinsing.

The performance stability of the prepared TFNC membranes was examined, as shown in Figure 16. All TFNC membranes developed from this work maintained good water permeance and high NaCl rejection, implying the good adherence of the film on the ENF supports. The performance and mechanical properties of the membrane developed from this work were also compared to other TFNC membranes, and the TFC membrane on PAN supports was prepared using the conventional phase inversion technique as previously published and summarized in Table 4.

The water permeance of TFC membranes fabricated using nanofiber supports clearly outperformed those made with phase inversion supports. Additionally, the membranes developed in this study exhibited a superior separation performance compared to those prepared from polyvinylidene fluoride (PVDF) ENF supports. The mechanical robustness of the TFNC membrane developed in this work also exceeds that of the membrane based on PVDF-ENF support. It is important to note that the overall mechanical strength of the prepared membrane is primarily derived from the backing support, which provides sufficient reinforcement during membrane testing. Additionally, the underlying mechanical properties of the PA thin selective layer are strongly dependent on the long-term integrity and durability of the nanofiber support layer. The results from tensile tests, traditionally employed to assess the mechanical properties of membranes in laboratory settings, do not fully capture the mechanical behavior under actual operational conditions. To fully understand the mechanical behavior of TFC membranes, it is necessary to look into the mechanical properties and deformation mechanisms of each layer. Comprehensive investigations such as biaxial testing under static and fatigue loading to simulate real operational conditions are recommended [70].

Among the various TFNC membranes compared, those incorporating polysulfone (PSU) ENF supports exhibited the highest water permeance and mechanical strength. This superior performance is likely due to the unique cross-hatched pattern of the ENF and the enhancement of the support’s hydrophilicity through polydopamine modification [69].

Our research, conducted at relatively low-pressure conditions (5 bar) and with modest salt concentrations, offers a preliminary assessment of the membranes’ effectiveness for desalination applications. The findings suggest their potential use in treating brackish water with salt concentrations between 2000 and 10,000 ppm and their applicability in the final stages of water purification, particularly at lower operating pressures. To verify their compatibility with reverse osmosis (RO) processes for seawater desalination, further testing under conditions that closely mirror the real operational environment is recommended.

## 4. Conclusions

Polyamide TFC membranes were successfully developed on an electrospun nanofiber support following subsequent treatment modification. In this work, the heat-pressed and alkaline hydrolysis treatment had important influences on the morphology and physicochemical properties of the nanofiber support layer, which subsequently governed the PA thin film’s structure–properties relationship during the TFNC membrane’s performance. The proper heat-pressed treatment in ENF support was found to be crucial for the formation of dense PA thin selective films. Increasing the heat-pressed temperature caused a reduction in the pressure-normalized flux due to a smaller support pore size. However, the TFNC membranes demonstrated improved pressure-normalized flux using hydrolyzed ENF as the support layer while maintaining outstanding NaCl rejection due to the increased amide and carboxyl group contents, leading to enhanced hydrophilicity support. The support pore size and hydrophilicity were revealed to play a major role in the enhancement of both selectivity and permeability. This superior improvement is believed to be due to the enhanced adhesive force between the PA thin film nanofiber support layers. Furthermore, rinsing with n-hexane after the IP reaction might boost the pressure-normalized flux by 29%. Finally, the TFNC membranes demonstrate an excellent future as an alternate choice in desalination, and further investigation into the membrane’s characteristics in separation performance is highly recommended.

## Figures and Tables

**Table 1:** Characteristics of the ENF support after thermal treatment at different temperatures.

Support	Fiber Diameter (nm)	Mean Flow Pore Pressure (bar)	Mean Pore Size (µm)
As-spun ENF	150 ± 30	0.51	1.26
hp-ENF 120	200 ± 30	0.70	0.91
hp-ENF 140	220 ± 40	0.79	0.81
hp-ENF 160	240 ± 60	1.02	0.63

**Table 2:** The properties of hp-ENF support before and after hydrolysis at different temperatures.

Hydrolysis Temperature (°C)	Fiber Diameter (nm)	Mean Pore Size (µm)	I_1665_/I_2242_	I_1565_/I_2242_	WCA (°)
Non-hydrolyzed	200 ± 30	0.91	0	0	25.5 ± 7.2
30	120 ± 20	0.71	0.157	0.055	0
50	120 ± 10	0.66	1.024	5.941	0

**Table 3:** Summary table of the properties of the TFNC membrane with different hydrolysis temperatures.

Hydrolysis Temperature (°C)	*n*-Hexane Rinsing	PA Layer Thickness (nm)	I_1446_/I_1541_ (FTIR)	O/N Ratio (XPS)	A (LMH bar^−1^)	R (%)
Non-hydrolyzed	no	700 ± 50	0.270	3.24	2.1 ± 0.7	83.0 ± 6.4
30	no	410 ± 50	0.299	1.94	2.0 ± 0.1	97.6 ± 1.0
30	yes	380 ± 40	0.309	1.85	2.9 ± 0.2	96.8 ± 0.4
50	no	430 ± 40	0.314	3.58	2.7 ± 0.0	98.0 ± 0.4

**Table 4:** Performance comparison and mechanical properties of TFC membranes.

Membranes	Feed Solution	Operating Pressure (bar)	A (LMH bar^−1^)	R (%)	Mechanical Properties		Ref
					Tensile Strength (MPa)	Strain at Break (%)	
hp-TFNC	NaCl (2000 ppm)	5	2.1 ± 0.7	83 ± 6.4	20.1 ± 4.1	27.1 ± 4.4	This work
hp-TFNC 120-30			2.0 ± 0.1	97.6 ± 1.0	16.3 ± 2.5	24.5 ± 3.7	This work
hp-TFNC 120-30-Hex			2.9 ± 0.2	96.8 ± 0.4	21.7 ± 1.0	27.5 ± 0.9	This work
hp-TFNC 120-50			2.7 ± 0.2	98.0 ± 0.4	17.6 ± 7.5	24.7 ± 0.9	This work
TFNC from PVDF	NaCl (1000 ppm)	8	1.9 ± 0.1	91.2 ± 1.3	5.4 ± 0.7	27.1 ± 0.9	[7]
TFNC from PAN/CA	NaCl (500 ppm)	7	2.8 ± 0.9	97.5 ± 0.4	N/A	N/A	[21]
TFNC from PSU	NaCl (2000 ppm)	20	5.5 ± 0.4	98.7 ± 0.1	40	3.5	[69]
TFC from hydrolyzed PAN prepared from phase inversion *	NaCl (5850 ppm)	10	0.91	89.95	N/A	N/A	[1]
TFC from support prepared from phase inversion	NaCl (2000 ppm)	15.5	0.6 ± 1.0	96.7 ± 1.4	N/A	N/A	[3]

* Tested with dead-end filtration mode. N/A is an abbreviation for not available.

## Data Availability

Data are contained within the article.

## Supplementary Materials

The following supporting information can be downloaded at: https://www.mdpi.com/article/10.3390/polym16050713/s1, Figure S1: SEM images of the backing support materials (a) without heat-pressing and with heat-pressing at (b) 120 °C and (c) 140 °C; Figure S2: WCA of hp-ENF 120 before (left) and after hydrolysis (right); Figure S3: (a) Photograph of peeled PA film from TFNC membrane on the double-sided tape (b) Photograph of peeled PA film from TFNC 50 membrane on the double-sided tape; Figure S4: The proposed mechanism of interaction between hydrolyzed PAN-co-MA ENF support and PA-selective film; Figure S5: (a) Wide-scan XPS spectra of TFNC 30 (b) O1s deconvolution of TFNC 30 with n-hexane rinse; Table S1: Pure water permeance (PWP) of backing support heat-pressing at different temperatures; Table S2: Weight loss of nanofiber support upon hydrolysis; Table S3: XPS elemental composition of O, N, C, O/N ratio and amide/carboxyl ratio of TFNC 30, (XPS depth measurement of 10 nm). Refs. [3,29,65,71] are cited in the supplementary materials.
