# Supplementary material for: Evaluating Post-Treatment Effects on Electrospun Nanofiber as a Support for Polyamide Thin-Film Formation
Source: Polymers (Basel). 2024 Mar 5;16(5):713. doi: 10.3390/polym16050713 (PMC10934950; doi:10.3390/polym16050713)
Supplement: Supplementary file 1 [file polymers-16-00713-s001.zip › polymers-2876276-supplementary.pdf]

polymers

MDPI

Article

Evaluating Post-Treatment Effects on Electrospun Nanofiber as a Support for Polyamide Thin-Film Formation

Anniza Cornelia Augusty [1], Ratthapol Rangkupan [2] and Chalida Klaysom [1,*]

[1] Center of Excellence in Particle and Material Processing Technology, Department of Chemical Engineering, Chulalongkorn University, Bangkok 10330, Thailand; annizagst@gmail.com
[2] Metallurgy and Materials Science Research Institute, Chulalongkorn University, Bangkok 10330, Thailand; ratthapol.r@chula.ac.th
* Correspondence: chalida.kl@chula.ac.th

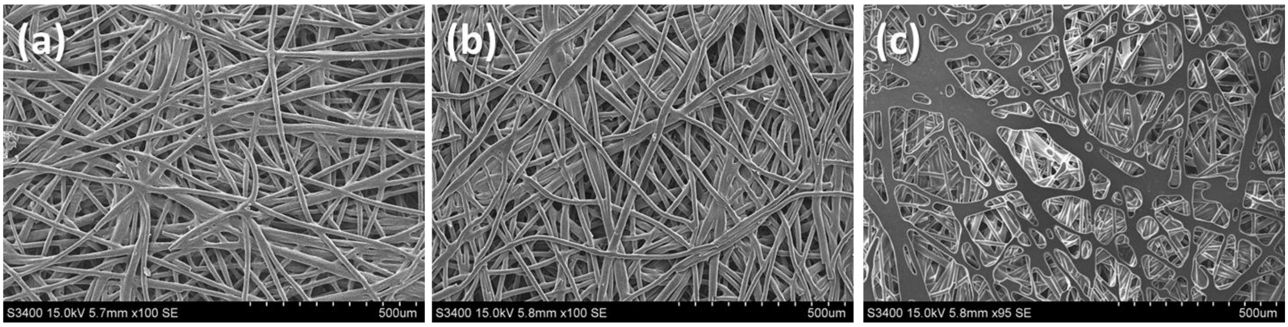

Figure S1. SEM images of the backing support materials (**a**) without heat-pressed and with heat-pressed at (**b**) 120 °C and (**c**) 140 °C.

The pure water permeance (PWP) of the ENF support with an effective area of 7.84 cm^2 was performed using a dead-end filtration cell pressurized with N_2 gas at 0.1 bar. The variation in PWP of the backing support, treated at different temperatures, is presented in Table S1. It was observed that the PWP of the backing support decreases as the heat treatment temperature increases. Specifically, the PWP of the backing support treated at 140°C experienced a significant reduction of 60%. This notable decrease commenced when the backing support began to partially melt, as evidenced in Figure S1.

Table S1. Pure water permeance (PWP) of backing support heat-pressed at different temperature

Backing support	PWP (LMH bar^{-1})
Without heat treatment	81,079 ± 1999
Heat treated at 120	72,124 ± 1189
Heat treated at 140	28,799 ± 434
Heat treated at 160	23,925 ± 825

Table S2. Weight loss of nanofibers support upon hydrolysis

Hydrolysis Temperature (°C)	Weight loss (%)
30	0.0
50	0.1

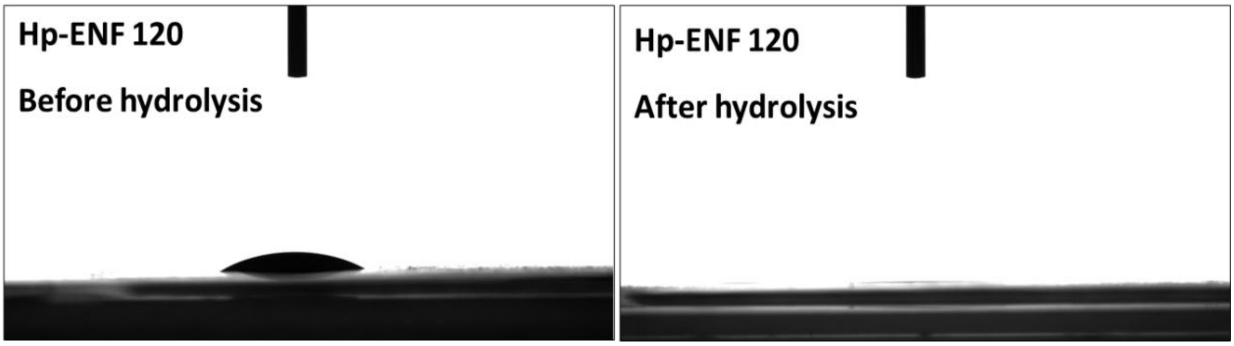

Figure S2. WCA of hp-ENF 120 before (**left**) and after hydrolysis (**right**).

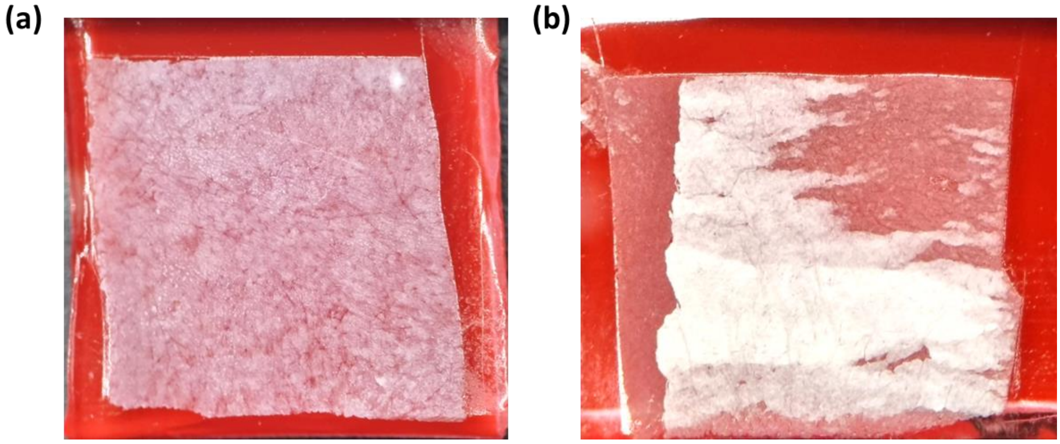

Figure S3. (**a**) Photograph of peeled PA film from TFNC membrane on the double-sided tape (**b**) Photograph of peeled PA film from TFNC 50 membrane on the double-sided tape.

From Figure S3a, it is clearly shown that the PA layer is peeled off from the ENF support of the TFNC membrane, showing the transparent region. In contrast, after being peeled off, some regions with more opaque and white color indicated that both PA layer and nanofiber support could detach from the backing support. It could be implied that the firm adherence of PA selective layer on the nanofiber support (Figure S3b) is strongly dependent of support properties.

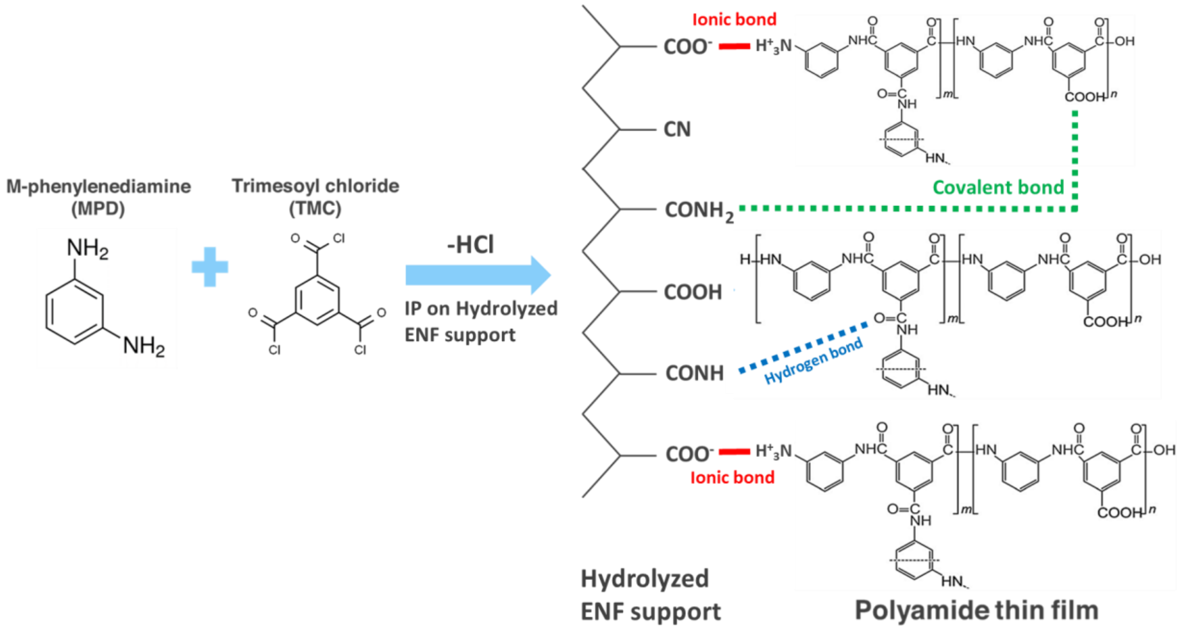

Figure S4. The proposed mechanism of interaction between hydrolyzed PAN-co-MA ENF support and PA selective film.

To better understand the mechanism of interaction between hydrolyzed ENF support and PA selective film, Figure S4 explains the schematic illustration of chemical structure and possible chemical bonding. The improved selectivity of TFNC membranes after using a hydrolyzed ENF support layer indicated the outstanding selective film attachment on the porous nanofiber support. The stronger hydrolysis degree after elevating the reaction temperature to 50 °C could generate an extensive amount of amide and carboxyl

groups, which play a significant role in the increase of support hydrophilicity and overall pressure-normalized flux of TFNC membranes. These amide groups from hydrolyzed ENF support could form electrostatic interaction via hydrogen bonding with another amide group from a cross-linked PA layer, as a result of IP reaction between MPD solution and TMC organic solution [1]. In addition, the carboxylic acid is ionized and deprotonated, facilitating the ease of forming ionic bonds with amino groups in polyamide chains [2, 3]. Besides these two chemical bonds, the establishment of a strong covalent bond possibly happens through the interaction between the amide bond from hydrolyzed ENF support and the linear COOH part of the PA thin film. In addition, the interaction between amide-containing hydrolyzed ENF support with some contents of acyl chloride in the TMC solution potentially forms the covalent bond accordingly [4]. As a result, the interfacial adhesion support-PA layers are becoming more robust, improving the membrane performances and stability over a long duration.

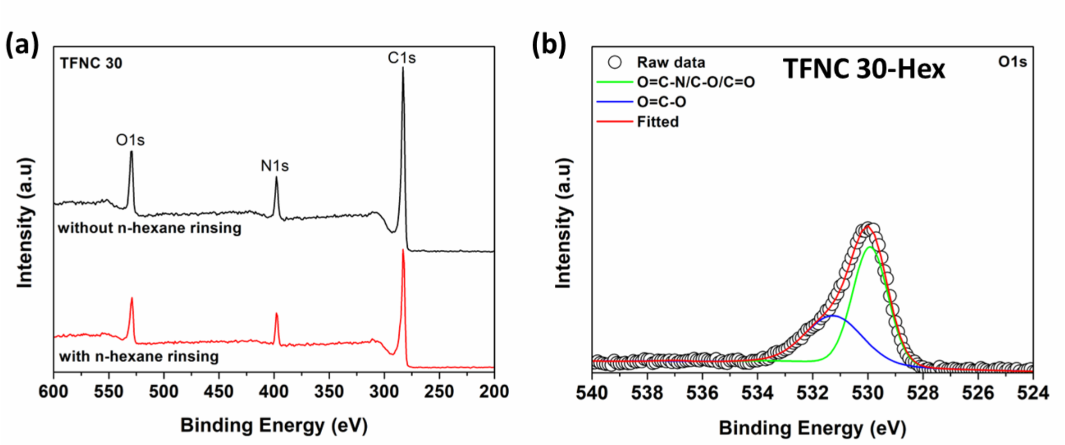

Figure S5. (**a**) Wide-scan XPS spectra of TFNC 30 (**b**) O1s deconvolution of TFNC 30 with n-hexane rinse.

Table S3. XPS elemental composition of O, N, C, O/N ratio and amide/carboxyl ratio of TFNC 30, (XPS depth measurement of 10 nm).

Treatment in PA film	O%	N%	C%	O/N ratio	O=C-N (%)	O=C-O (%)	O=C-N/O=C-O ratio
Without n-hexane rinse	24.26	12.49	63.25	1.94	59.36	40.64	1.46
With n-hexane rinse	24.5	13.27	62.23	1.85	61.23	38.77	1.58

XPS characterization of TFNC membranes prepared on hydrolyzed ENF support hydrolyzed at 30 °C, followed by n-hexane rinse after the interfacial polymerization reaction was demonstrated in Figure S5 and the XPS elemental composition was shown in Table S3.

References

1. Oh, N.-W., J. Jegal, and K.-H. Lee, *Preparation and characterization of nanofiltration composite membranes using polyacrylonitrile (PAN). II. Preparation and characterization of polyamide composite membranes.* Journal of Applied Polymer Science, 2001. **80**(14): p. 2729-2736.
2. Park, S.-J., et al., *Fabrication of polyamide thin film composite reverse osmosis membranes via support-free interfacial polymerization.* Journal of Membrane Science, 2017. **526**: p. 52-59.
3. Huang, Y.-H., et al., *A study on the characteristics and pervaporation performance of polyamide thin-film composite membranes with modified polyacrylonitrile as substrate for bioethanol dehydration.* Polymer International, 2014. **63**(8): p. 1478-1486.
4. Geng, C., et al., *Enhancing the long-term separation stability of TFC membrane by the covalent bond between synthetic amino-substituted polyethersulfone substrate and polyamide layer.* Journal of Membrane Science, 2021. **637**.
